# Further evidence needed to change policy for the safe and effective radical cure of vivax malaria: Insights from the 2019 annual APMEN Vivax Working Group meeting

**DOI:** 10.1002/app5.314

**Published:** 2021-01-12

**Authors:** Varunika Sonani Hapuwatte Ruwanpura, Spike Nowak, Emily Gerth‐Guyette, Minerva Theodora, Lek Dysoley, Mebratom Haile, Koen Peeters Grietens, Ric Norman Price, Caroline Anita Lynch, Kamala Thriemer

**Affiliations:** ^1^ Global and Tropical Health Division Menzies School of Health Research and Charles Darwin University Darwin Australia; ^2^ PATH Seattle WA USA; ^3^ National Malaria Control Program of Indonesia Indonesia; ^4^ National Centre for Parasitology, Entomology and Malaria Control Cambodia; ^5^ School of Public Health National Institute of Public Health Cambodia; ^6^ National Malaria Control and Elimination Program, Disease Prevention and Control Directorate Federal Ministry of Health Ethiopia; ^7^ Institute of Tropical Medicine Antwerp Belgium; ^8^ School of Tropical Medicine and Global Health Nagasaki University Nagasaki Japan; ^9^ Mahidol‐Oxford Tropical Medicine Research Unit, Faculty of Tropical Medicine Mahidol University Bangkok Thailand; ^10^ Centre for Tropical Medicine and Global Health, Nuffield Department of Clinical Medicine University of Oxford Oxford UK; ^11^ Medicines for Malaria Venture Geneva Switzerland; ^12^ Faculty of Epidemiology and Population Health London School of Tropical Medicine and Hygiene London UK

**Keywords:** Asia Pacific, evidence gaps, malaria elimination, malaria health policy, *Plasmodium vivax* malaria, policy implementation, radical cure

## Abstract

New diagnostics and treatment options for the radical cure of *Plasmodium vivax* malaria are now available. At the 2019 annual meeting of the Vivax Working Group of the Asia Pacific Malaria Elimination Network, participants took part in a roundtable discussion to identify further evidence required to introduce these new tools into policy and practice. Key gaps identified were accuracy and reliability of glucose‐6‐phosphate‐dehydrogenase deficiency tests, health system capacity, and feasibility and cost effectiveness of novel treatment strategies in routine clinical practice. As expected, there were differences in the priorities between country partners and researcher partners. To achieve the 2030 target for the regional elimination of malaria, evidence to address these issues should be generated as a matter of priority. Review of global guidelines alongside locally generated data will help to ensure the timely revision and optimisation of national treatment guidelines that will be vital to meet regional elimination goals.

## 1 INTRODUCTION

*Plasmodium vivax* malaria continues to exert huge public health burden in the Asia‐Pacific region, South America and the Horn of Africa with more than 14.3 million clinical cases in 2017 (Battle et al., 2019). Major gains have been made in reducing this burden that reflects, in part, sustained global attention to malaria and renewed attention to the United Nations' Sustainable Development Goals (SDG). However, these successes in disease control are often focal in nature, vulnerable to reversal and consequently threaten national, regional and international health security. In 2014, governments in the Asia‐Pacific region committed to eliminating malaria by 2030 (Department of Foreign Affairs and Trade, 2014). Until recently, efforts to control and eliminate *P. vivax* malaria were limited by the availability of effective radical cure options.

Currently, the treatment for *P. vivax* malaria recommended in most countries in the Asia‐Pacific and the Horn of Africa is a combination of a blood schizonticide such as chloroquine (CQ) or an artemisinin combination therapy (ACT) plus a 14‐day course of primaquine (PQ) to kill the dormant liver stages called hypnozoites (World Health Organization [WHO], 2015b). In these regions, a supervised course of PQ14 (at a dose of 3.5 mg/kg total dose) has variable efficacy—achieving greater than 70% at 6 months in some, but not all locations (John et al., 2012; Llanos‐Cuentas et al., 2019). However, in reality, there are several challenges including the lack of implementation of radical cure policies (Recht et al., 2018) resulting in low prescription rates often due to concerns about haemolysis in patients with glucose‐phosphate‐dehydrogenase deficiency (G6PD) (Ley et al., 2017). But even if policies are implemented, the low adherence to a prolonged course of treatment results in lower effectiveness (Abreha et al., 2017; Douglas et al., 2017; Ley et al., 2017).

While the burden of malaria continues to fall across the Asia‐Pacific, the proportionate burden of *P. vivax* malaria cases has increased, highlighting the importance of improving the radical cure of malaria to achieve the 2030 target for regional elimination (APMEN, 2019). The recently published Lancet Commission highlights the need and feasibility of malaria elimination but also emphasises the importance of accelerating the introduction of novel strategies (Feachem et al., 2019). In the last 12 months, there have been three major advances in the tools available to tackle *P. vivax* relapses: (i) a short course high daily dose PQ regimen (Taylor et al., 2019); (ii) a novel quantitative point‐of‐care G6DP test (Alam et al., 2018; Pal et al., 2019); and (iii) single dose Tafenoquine (TQ) (Lacerda et al., 2019; Llanos‐Cuentas et al., 2019). While broad global policy recommendations addressing the use of those tools are expected from the World Health Organization's (WHO) Global Malaria Programme (GMP) within the next 1 or 2 years, the adoption of new tools will need to be tailored to individual country needs and capacities. After 60 years of limited biotechnical innovation for the control of *P. vivax* malaria, National Malaria Control Programs (NMCPs) are now faced with the challenge of deciding between several new strategies. Options include, but are not limited to, improving adherence to current guidelines, introducing quantitative or qualitative G6PD testing with current treatment, shorter high dose PQ regimens and TQ with quantitative G6PD testing. Introduced strategies may include a mix of these options at different levels of the health system to be rolled out simultaneously or as phased implementation depending on the country context and capacity.

Considering that globally the estimated time between the availability of scientific evidence and its introduction into clinical practice is approximately 17 years (Balas & Boren, 2000; Green et al., 2009; Morris et al., 2011; Trochim, 2010; Westfall et al., 2007), we aimed to bring policymakers together to bridge this gap at the same time that NMCPs are preparing revisions of their National Strategic Plans (NSP) and Global Fund (GF) concept notes. Previous experience with policy changes and their implementation for the treatment of falciparum malaria further highlights the need to institutionalise national malaria policy review and improve communication regarding treatment policy at all levels (Amin et al., 2007; Kamya et al., 2002; Mulligan et al., 2006; Shretta et al., 2000; Williams et al., 2004). Key recommendations suggested are to improve communications from government to health worker level to support implementation, inclusion of pharmaceutical companies in the process to ensure timely registration of novel drugs, active lobbying by the international community to ensure funding for novel tools at country level and to ensure that countries address national level questions in a timely manner (Kshirsagar, 2006; Williams et al., 2004).

NMCPs and national policymakers must consider several factors when revising treatment policies including the available scientific evidence, WHO recommendations, country‐specific considerations regarding malaria epidemiology and G6PD deficiency (including in‐country heterogeneity), local socio‐ecological variations, cost‐effectiveness, logistics and health system capacity. Countries' national planning cycles include National Strategic Plans (NSP), Mid Term Reviews (MTR) and Malaria Programme Reviews (MPRs) which again affect the timely approval of new policy. In addition, many NMCPs rely on external funding from organisations such as the GF that influence decision‐making and timelines at country level. The GF, in turn, relies on WHO prequalification and policy guidance for new tools at the global level. Factors influencing the uptake of these tools include NMCP or Ministry of Health perceptions of ownership of research (countrywide and on a larger scale), the timeliness at which evidence becomes available, the reliability of research data, available funding and cost‐effectiveness (Nutley et al., 2007; Peters, 2018; Smith, 2013). Finally, the incentive to implement the process of policy change can be driven by national goals such as the target for malaria elimination.

In 2019, the Asia Pacific Malaria Elimination Network's (APMEN) Vivax Working Group (VxWG) convened its annual meeting in Kathmandu, Nepal, and was attended by over 140 participants. Roundtable discussions and a workshop were held to identify evidence gaps and potential research questions pertinent to NMCPs' policy change processes in a timely manner. The aim was to identify these questions as NMCPs are preparing revisions of their NSPs and GF concept notes. While countries await the revised WHO global recommendations, which are anticipated by mid‐2021, NMCPs and regional policymakers need to consider the additional information required to initiate country‐level changes once these recommendations are in place.

This article explores the additional evidence required by NMCPs that aim to initiate policy change to implement safe and more effective radical cure for *P. vivax* malaria. It highlights the common issues faced, as well as country‐specific questions that need to be addressed. The aim of this work was to facilitate an NMCP‐guided translational research agenda for the short to medium term to assist the decision‐making processes for the potential introduction of novel radical cure strategies.

## 2 METHODS

### 2.1 The Vivax Working Group of the Asia Pacific Malaria Elimination Network (APMEN)

The VxWG of the APMEN was formed in 2009 at the network's inaugural meeting. *P. vivax* was identified as a critical challenge for regional malaria elimination. The group now comprises representatives from 21 NMCPs, research partners, the WHO, as well as key funding, global health and industry stakeholders. Together with the Asia Pacific Leaders Malaria Alliance (APLMA), APMEN forms a collaborative network working to overcome the challenges to eliminating malaria in the Asia‐Pacific region (Thriemer et al., 2017, 2018; Vivax Working Group, 2015). Over the last 9 years, the VxWG has fostered dynamic interactions between stakeholders to guide the development and optimisation of tools and products for the diagnosis and treatment of *P. vivax* . With the availability of novel tools (Alam et al., 2018; Lacerda et al., 2019; Llanos‐Cuentas et al., 2019; Pal et al., 2019; Taylor et al., 2019), the time has now come to shift the focus from product development to the implementation of these tools into policy and practice.

### 2.2 Participant characteristics

All participants taking part in the main annual meeting were included in the roundtable discussion and were categorised into two groups: country partners (CPs) comprising delegates from NMCPs, and research partners (RPs). RPs included researchers from endemic countries and non‐endemic countries, Civil Society Organisations (CSOs), Product Development Partnerships (PDPs), manufacturers, donors, WHO, APMEN and APLMA. During the consequent workshop, only CPs participated. Three post‐meeting interview CP participants were selected based on their availability in the weeks after the meeting and all three interviewees were present during both the main meeting and the workshop.

### 2.3 Data collection

Data was collected in roundtable discussions at the annual meeting, in a consequent CP workshop and in follow‐up interviews with select CPs who attended the annual meeting.

#### 2.3.1 Roundtable discussions

During the main meeting, a roundtable discussion was held, focusing on defining the evidence gaps relevant for policy change. The aim of these discussions was to initiate dialogue around what evidence was required to implement national level policy change for adopting novel radical cure options. Conference attendees were allocated to separate tables for CPs and RPs to ensure opinions were captured differentially. CP table allocation was done across subregions to encourage diversity of opinions on each table. In the case of the CPs, there were three tables with 12–14 participants each. RPs were represented in six tables with 10–11 participants each. Each table had a facilitator and a note taker to ensure that table discussions were focused, and discussion outcomes were recorded accurately (Appendix A). Prior to the meeting, facilitators and note takers were briefed on the key objectives of the discussions. At the end of the discussions, the CP tables presented their discussion outcomes to the plenary. Outcomes from the RP discussion were collated separately. Notes and outcome summaries were used for data analysis.

#### 2.3.2 Workshop

Following the initial two‐day meeting, a workshop restricted to CPs was held to explore the additional evidence required by each country to develop a road map for policy change and implementation. CPs were divided into nine discussion tables (with 2–3 countries per table) and each table was allocated a facilitator and note taker. Workshop discussions were based on the assumption that a WHO recommendation was available for high dose short course PQ and TQ. Directly after the end of the roundtable discussions of the main meeting and in a consultative process with the facilitators, the authors distilled nine research questions relevant for policy change. The questions were written on flipcharts and participants were asked to rank them by priority.

#### 2.3.3 Follow‐up interviews

Follow‐up Skype or email semi‐structured interviews were held with three CPs—Ethiopia, Indonesia and Cambodia—in November and December 2019. The objectives of these additional interviews were to verify the priority research questions ranked during the workshop, investigate if any further evidence gaps had been identified after the workshop and understand if further discussion with other NMCP members may have altered priority questions.

### 2.4 Data analysis

Our approach to analysis was qualitative and semi‐quantitative, as follows:

#### 2.4.1 Group discussion analysis

Notes from discussions of both CPs and RPs were analysed to identify research topics that both groups perceived as important for policy change. To further understand the relative importance of each research topic to the two groups, all notes were combined, and a count made of the frequency with which each of the emergent research topics were discussed—we refer to these as research topic frequency scores.

In addition, all notes for CPs and RPs were combined and a word cloud generated to conceptualise emerging major themes—this consisted of a visual representation of text data with words weighted by their frequency of use in a document. The word cloud was created using the online software tool http://www.wordclouds.com by merging all group work notes, removing country names, correcting spelling, and checking frequently used words for variances in how they were expressed (for instance PQ‐14 and 14‐day PQ were both expressed as PQ14). Once the word cloud was generated, the frequent word list was double‐checked for duplicated words with incorrect spelling or similar meanings.

#### 2.4.2 Prioritisation of research topics

During the workshop, facilitators developed a list of research topics that emerged from the roundtable discussions of both CPs and RPs. A total of nine topics areas were developed. During the workshop, CPs were asked to prioritise research questions they perceived as important for policy change by ranking them from a score of 1 (the highest priority) to 5 (the lowest). Questions which were not ranked by participants were given a score of six. The average country priority ranking for each research question was then calculated to identify the most important research questions.

#### 2.4.3 Semi‐structured interviews

One interview was conducted via Skype and notes were taken, and the two other interviews were done via email. All transcripts were manually coded for confirmed priority areas, novel priority areas, potential other opinions and future actions. Passages of text were read, and codes assigned based on themes, and examined for frequency, trends, similarities, and differences. When necessary, direct speech in the results section was corrected for grammatical errors to improve readability; words added by investigators to improve clarity were marked by square brackets.

#### 2.4.4 Comparison of country partner and research partner priorities

We compared RP and CP priority topics by assessing research priority frequency scores from group discussions. As there were twice the number of RPs than CPs during discussion groups, we incorporated CP ranking of research topics into the CP frequency scores by multiplying the original frequency score by average rank scored. We adapted lessons learned expressed in Williams et al. (2004) to develop a continuum of policy change along which we plotted research topic frequency scores. Four key phases were identified up to policy change: (i) current implementation; (ii) clinical studies to guide global policy; (iii) national adaptation of new tools; and (iv) national policy change to new tools. Research topics related to current practice were included under ‘current implementation’. Efficacy trials were included under ‘clinical studies’ and ‘national adaptation of new tools’ included feasibility and effectiveness studies—or any studies that would allow CPs to assess the use of new tools within their respective health systems. ‘National policy change’ included elements such as translation of evidence and coordination between RPs and CPs. The placement of circles for research topics along the continuum of change was done by two authors and agreed through follow‐up discussions. Topics were represented by circles, the size of which was related to the frequency score with larger circles representing higher scores.

## 3 RESULTS

### 3.1 Participants

A total of 43 CPs and 97 RPs attended the meeting (Appendix B and Appendix C). CP representation within the Asia‐Pacific region included participants from Afghanistan (n = 2), Bangladesh (n = 1), Bhutan (n = 3), Cambodia (n = 2), China (n = 1), India (n = 2), Indonesia (n = 2), Lao (n = 3), Malaysia (n = 2), Nepal (n = 10), Pakistan (n = 1), the Philippines (n = 1), Papua New Guinea (PNG) (n = 1), Republic of Korea (n = 2), Solomon Islands (n = 2), Sri Lanka (n = 2), Thailand (n = 2), Vanuatu (n = 1) and Vietnam (n = 2). An additional observer from Ethiopia (n = 1) also attended. Among the 97 RPs there were 39 (39.8%) researchers from endemic countries, 24 (24.7%) researchers from non‐endemic countries, 7 (7.2%) representatives from CSOs, 10 (10.3%) delegates from PDPs, 3 (3.1%) manufacturers, 1 (1.0%) international WHO representative and 2 (2.1%) officials from the WHO office in Nepal, 3 (3.1%) APMEN representatives, 3 (3.1%) APLMA officials and 3 (3.1%) donor representatives. Most researchers from both endemic and non‐endemic countries had a biomedical research focus including clinical and lab science or modelling and statistics skills (92.1%; 58/63) and 5 (7.9%) participants worked in the field of social science or had a health economics focus.

### 3.2 Key research questions identified by country partners during main meeting

The most frequent topics identified as being key to inform policy change and implementation were cost‐effectiveness and cost–benefit, understanding policy change processes, operational feasibility and health system capacity, G6PD test accuracy and reliability, treatment efficacy and G6PD prevalence. In addition, safety, drug acceptability, treatment effectiveness and adherence and *P. vivax* prevalence were also ranked as important (Appendix D and Appendix E).

### 3.3 Key research questions identified by research partners during main meeting

**FIGURE 1:**
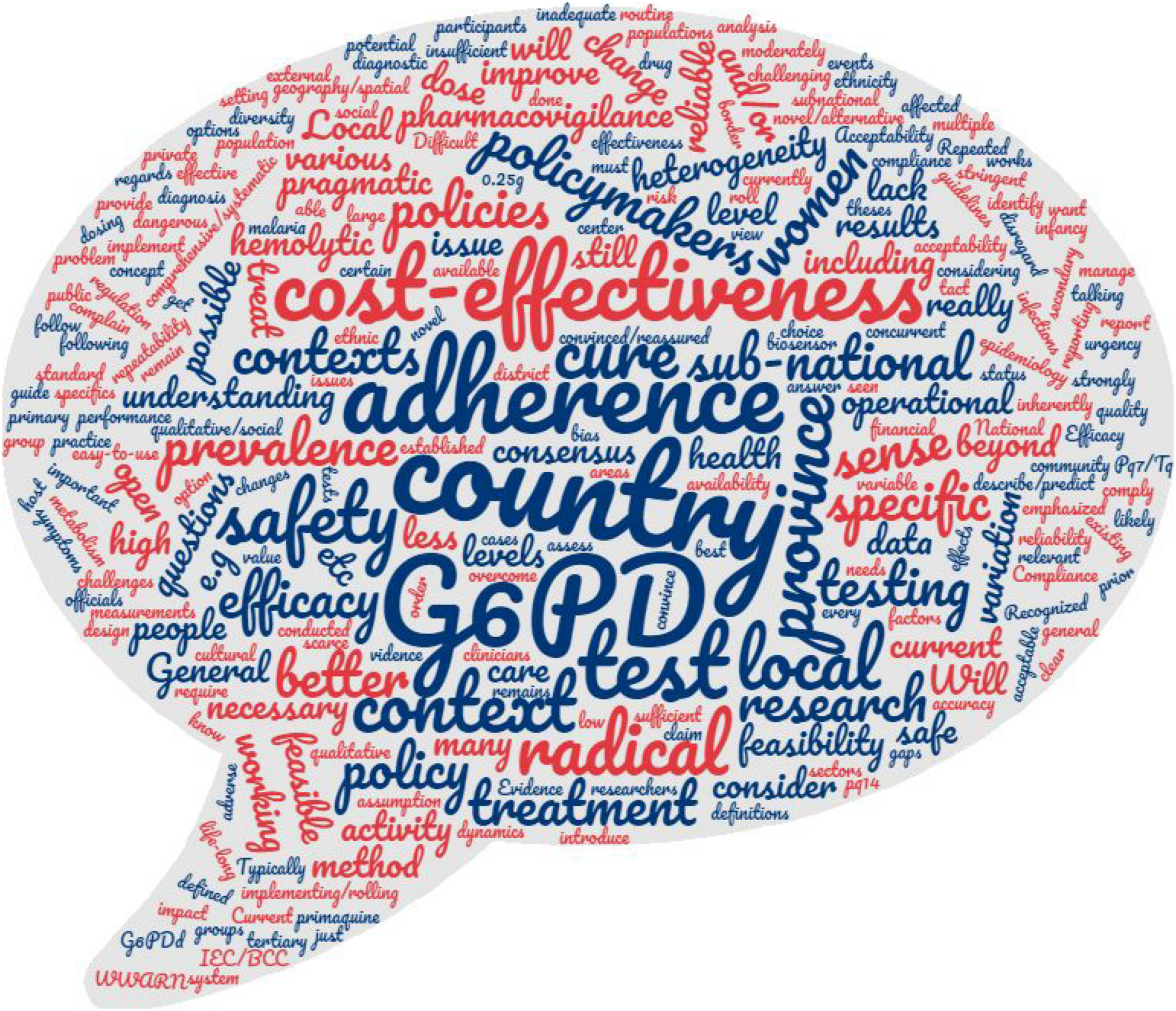
Word cloud identifying key topics from research partner discussions

Research areas, questions and topics identified by the six RP tables during the meeting encompassed a wide variety of issues. Overarching topics included the safety and appropriate testing for G6PD, effectiveness and adherence of radical cure options, operational feasibility and health care system capacity (Table 1 and Figure 1). RPs also highlighted other challenges, beyond research, that require better understanding or strengthening such as variations in government regulation of the public and private health sector in different APMEN countries that affects policy regulation, revision and implementation. Understanding *P. vivax* epidemiology and associated mortality to facilitate advocacy as well as the need to improve communication with patients to highlight the benefits of radical cure were also mentioned.

**TABLE 1:** Evidence gaps and research questions identified by research partners

Overarching topics	Identified evidence gaps and research questions
Safety and appropriate testing for G6PD	• How good is the reliability and field applicability of G6PD tests? • Is a single time point to test for G6PD activity sufficient (i.e., one life‐long diagnosis)? • How to conduct effective training G6PD testing including QC? • What is the more comprehensive picture on G6PD deficiency levels across different populations? • Country‐specific data on the adverse effects of PQ and TQ is required as well as better definitions to describe, predict and manage haemolytic events
Effectiveness and adherence of radical cure options	• Lack of comparative effectiveness data between PQ7 and PQ14 and TQ • Limited evidence to support country claims that PQ14 is working • In which areas is low dose PQ with increased adherence (supervision) sufficient? • Pragmatic qualitative studies on adherence
Feasibility and health care system capacity	• Need for large‐scale implementation and feasibility studies particularly in large countries such as Indonesia and India with many ethnic groups • Cost‐effectiveness on subnational level • Pharmacovigilance strengthening • Feasibility of implementation of different radical cure options on primary, secondary, and tertiary health care levels • Operational challenges for roll out of easy‐to‐use, safe and reliable G6PD testing

### 3.4 Prioritisation of questions by country partners during the workshop

To clarify which of these topics had the highest priority for CPs, nine key questions were distilled after the initial discussions at the main meeting. These included: (i) What is the effectiveness of different treatment options (14 day low dose PQ (PQ14), 7 day high dose PQ (PQ7), TQ) and adherence between different regimens? (ii) What are the overall vivax dynamics in my country? (iii) How effective is current practice? (iv) What is the prevalence of G6PD deficiency in my country? (v) How well do new diagnostics work in the field (usability, robustness, etc.)? (vi) How can we ensure safe delivery of the different options? (vii) What is the cost effectiveness of the different regimens? (viii) Feasibility of new interventions at different levels of health system (including supply chain capacity and quality assurance)? (ix) What is the best way to improve patients' adherence?

Of these nine research questions, CPs ranked effectiveness of different radical cure options as the highest priority followed by questions around how well G6PD diagnostics work in the field. Evaluating methods to improve adherence was ranked as the third priority. Cost‐effectiveness of different options and feasibility as well as more data on G6PD prevalence within countries were ranked equally following the initial three priorities (Table 2). There were significant differences in rankings by country (Appendix F).

**TABLE 2:** Average ranking of country research priorities, with 1 being highest priority and 5 lowest (no priority = score of 6)

Question	Average rank
What is the effectiveness of different treatment options (PQ14, PQ7, TQ) and adherence between different regimens?	**2.6**
How well do new diagnostics work in the field (usability, robustness, etc.)?	**3.9**
What is the best way to improve patients' adherence?	**4.1**
What is the cost effectiveness of different regimen options?	**4.4**
Feasibility of new interventions at different levels of health system (including supply chain capacity and quality assurance)?	**4.4**
What is the prevalence of G6PD deficiency in my country?	**4.4**
How can we ensure safe delivery of the different options?	**4.9**
How effective is the current practice?	**5.3**
What are the overall vivax dynamics in my country?	**5.6**

### 3.5 Validation of country partner priority research topics

#### 3.5.1 Confirmation of priority ranking

All three CP participants interviewed confirmed that the priorities developed during the APMEN VxWG workshop remain priorities even after discussion with other NMCP members.

The efficacy and effectiveness of PQ7 remained the top‐ranking question for Cambodia:

We don't know about the efficacy of PQ treatment plans, but we think that 7‐day dose might be appropriate, and an increased dose might be effective, based on what other APMEN countries are doing also. (Cambodian NMCP representative)

The Indonesian NMCP representative confirmed that feasibility studies are their priority whereas the Ethiopian representative highlighted that operational feasibility of novel tools and cost effectiveness of G6PD testing at community level were the most pressing questions to address for their country as well as assessing gaps in current practice.

We need to assess whether new policy related to vivax radical cure could be implemented in a routine situation. (Indonesian NMCP representative)

It is good first to have conclusive evidence that shows the current practice has adherence issues. If not, the decision might be to continue with PQ14 to be on the safe side. (Ethiopian NMCP representative)

#### 3.5.2 Other opinions within the NMCPs

The Cambodian and Indonesian respondents reported that other NMCP colleagues and their respective Ministries of Health are also in agreement that these are each of their countries' key priorities. The Indonesian NMCP discussed these priorities with their national expert committee on October 24, 2019 after the 2019 APMEN meeting and before the interview, suggesting that there is a reasonable consensus within this expert community. However, the Indonesian respondent mentioned that there might be other opinions with diverging priorities at a higher Ministerial level. The Ethiopian respondent highlighted the need to reach full consensus among decision‐makers and that more discussions were needed within the country.

#### 3.5.3 Future action

In line with the identified priorities, Cambodia planned pilot studies to inform potential policy change.

Cambodia will only implement evidence‐based policy. We plan to investigate PQ7 and we would also like to explore how to implement TQ starting in the military. (Cambodian NMCP representative)

Similarly, feasibility studies were planned in Indonesia and in Ethiopia to generate data to support decision‐making.

TQ is not a priority at this point, we have huge amount of *P. vivax* . So, to minimise any unintended consequence as a result of this drug, we have to wait for some time [and gather more evidence]. (Ethiopian NMCP representative)

For Indonesia and Ethiopia, respondents mentioned that this did not yet influence national planning whereas in Cambodia these plans were closely linked to the currently developed NSP and these activities will be included in an upcoming application to the GF.

### 3.6 Research priorities on the continuum of change

**FIGURE 2:**
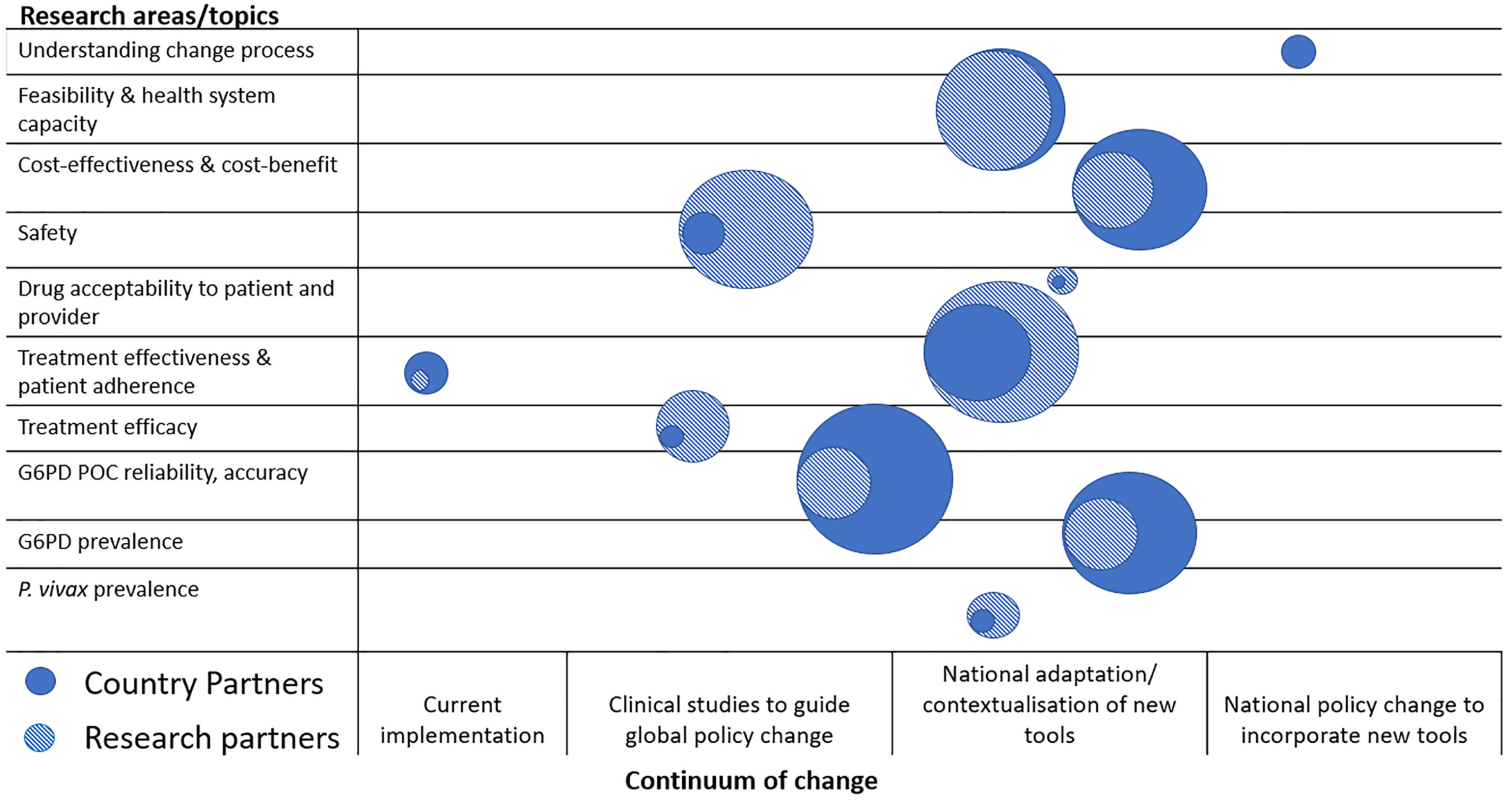
Research areas/topic priorities for country and research partners plotted along the continuum of change. Notes: Topics identified in the main meeting and the workshop are represented by circles. Circle sizes represent the frequency of topic discussed, with larger circles representing higher frequency

By combining CP topics and ranking into frequency scores, the highest priority topics raised by CPs became the use and reliability of point‐of‐care (PoC) G6PD tests on a par with cost‐effectiveness of new tools, G6PD prevalence, and feasibility and health system capacity, closely followed by patient adherence and effectiveness of novel treatment options (Figure 2). CPs emphasised costing, budget impact and cost–benefit analysis as well as cost‐effectiveness. Lower ranked priorities were *P. vivax* prevalence, current treatment effectiveness, understanding change processes, drug acceptability and safety. RPs prioritised patient adherence and effectiveness, safety, and feasibility and health system capacity. Additional priorities that were ranked lower were drug acceptability and cost‐effectiveness. The lowest ranked topic by RPs was *P. vivax* prevalence.

The greatest overlap between CPs and RPs was the need to understand the operational feasibility of implementing new tools in country health systems which was similarly ranked between CPs and RPs. Treatment effectiveness and patient adherence was the next greatest overlap followed by cost effectiveness and cost–benefit of different treatments. The greatest divergence between partners was regarding G6PD PoC accuracy and reliability, safety, and cost‐effectiveness. CPs highlighted the need to understand policy processes including how to better increase uptake of evidence into policy. RPs tended to highlight the need to map *P. vivax* prevalence or mortality to highlight to policymakers the need for vivax to be prioritised. Finally, research topics of interest to both CPs and RPs clustered around the national adaptation or contextualisation of new tools with CPs highlighting the need to understand policy processes or how to translate results from studies into guidance (Figure 2).

## 4 DISCUSSION

*P. vivax* malaria continues to be a major global public health threat. In the Asia‐Pacific region and the Horn of Africa, the highest caseloads are often in remote communities where access to healthcare is poor. With the recent approval of and evidence for the efficacy of new tools for better radical cure (Lacerda et al., 2019; Llanos‐Cuentas et al., 2019; Taylor et al., 2019; White, 2019), discussions were facilitated to identify key research questions faced by CPs seeking to adapt new tools into their respective settings. The overall results of our analysis highlight a call by CPs for additional national‐level evidence around PoC G6PD accuracy and reliability, operational feasibility of radical cure options within health systems and cost‐effectiveness analysis of new tools. This work highlights key questions CPs will face when aiming to change national policy and proposes areas which researchers and donors can focus their work and resources on. It indicates the need for an increased understanding of treatment effectiveness under real‐life conditions as compared to efficacy which is measured in carefully controlled settings (Thriemer et al., 2017). This is in line with questions faced by CPs around a national level understanding of how to incorporate these new tools into clinical practice. Previous work on malaria treatment policy change highlights the importance of undertaking effectiveness studies as well as efficacy studies to drive policy change (Durrheim & William, 2005). Countries need to contextualise research findings and global policy recommendations to suit their countries' socio‐economic needs and specific epidemiological contexts (Davis & Walker, 2019). Those studies are crucial to support policy change and ‘bridge the "know–do" gap’ as highlighted previously (WHO, 2005). Health policy change is complex requiring different types of evidence along the continuum of change (Lewin et al., 2012; Peters & Bennett, 2012). Malaria programs have the benefit of receiving global guidance from the WHO that collates and assesses global clinical trial and safety data for new treatments. However, WHO guidance is global by necessity thus early country adopters of new tools often need to determine whether and how these tools will work in their local contexts.

The need for greater understanding of PoC G6PD accuracy and reliability prior to policy change was prioritised by CPs and RPs indicating the need to pilot new G6PD diagnostic tools especially before implementing TQ or high dose PQ regimens. Both CPs and RPs expressed concerns regarding drug tolerability and safety. However, in this context, CPs seem to express this as ‘wanting to better understand the reliability of G6PD tests’ to ensure that these tools will correctly identify G6PD deficient (or intermediate) patients when used in tandem with higher dose regimens. Historically, the availability of G6PD PoC tests has been very limited. NMCPs' exposure to quantitative tests, which are now in the process of more widespread commercialisation and availability, has been limited but concerted efforts have been undertaken by partners to raise awareness of these products and how they can be used to support malaria control and elimination goals (Ley et al., 2015). The difference in the level of priority given to PoC G6PD by RPs and CPs likely reflects an asymmetry in information between the two groups possibly indicating the need for better knowledge transfer through synthesis and accessible presentation of G6PD literature as well as further studies to answer CPs' questions. Discussions also highlighted the need to have a broader understanding about safety of radical cure away from a focus on adequate testing only to a more holistic approach, including adequate detection of early signs of haemolysis and risk mitigation strategies.

While both CPs and RPs expressed the need for feasibility studies, there was little discussion as to what exactly should be included in these studies. RPs highlighted the need to understand how pharmacovigilance systems could be strengthened down to the lowest levels where new tools could be used. This could indicate that feasibility for CPs and RPs means understanding how new tools will be deployed within the health system. Previous qualitative research to understand the processes and timelines for the introduction of ACTs and mandatory confirmation of malaria prior to treatment (Ajayi et al., 2008; Swana et al., 2016) indicates that exploratory studies provide useful and effective pre‐implementation data. Other health system elements highlighted from previous research on malaria treatment policy change pathways include increasing the uptake of integrated community case management (iCCM) (WHO, 2018) and strengthening the health workforce (WHO, 2015a) to manage the use of novel tools. This begs the question as to how and at what cost can countries provide adequate supportive supervision to their health workforce to ensure effective and safe radical cure, particularly when scaled up.

Cost‐effectiveness studies on new tools and treatment regimens were also raised by CPs and RPs. Some countries require Cost‐Effectiveness Analyses (CEA) to allow the incorporation of new tools into insurance schemes (Escribano Ferrer et al., 2017; Hansen et al., 2017). In the APMEN region, China, Malaysia, Philippines and Thailand have a requirement for Health Technology Assessments (HTA) of new tools (Sivalal, 2009) akin to the NICE process in the United Kingdom (NICE, 2019), but this is not a requirement in most regional countries. The HTA requirement is likely correlated to the proportion of the malaria programs supported by domestic versus external funding. Some countries are more reliant on GF for procurement of tools which need to be WHO approved or prequalified prior to GF procurement (Pigott et al., 2012). Others have substantial domestic budgets that may require different sets of evidence or information for policy change (Pigott et al., 2012). While limited, some CPs discussed the need for cost–benefit analysis and budget impact studies in addition to cost‐effectiveness. This likely reflects their cognizance of having to advocate for additional resources within their respective ministries of health, providing cost data in different ways to do so.

Understanding policy change processes and uptake of evidence was also raised. CPs requested more and better translation of evidence into policy especially by neutral or non‐technical parties who could translate evidence into simple language. In addition, they specified the need for stronger coordination with researchers and better understanding of regulatory and policy change pathways (see Figure 2). The gap between scientific recommendations based on research findings and countries' motivations to change policy is often difficult to bridge (Smith, 2013). CPs' requests align with findings from Smith's review of knowledge transfer in which an increase in the use of research into policy and practice explored the following strategies: ‘ensuring research is accessible’, ‘developing ongoing, collaborative relationships’, ‘improving structural communication channels’ and ensuring that there are sufficiently high incentives among researchers and research users to engage in knowledge exchange (Smith, 2013, p. 21).

CPs were also keenly aware of their implementation needs and unsurprisingly were concerned about the success of current treatment practice which will need to continue beyond feasibility studies while countries await global WHO guidance. In addition, G6PD deficient patients will continue to receive either the current standard treatment or 8‐week PQ. Understanding current adherence rates and reasons for non‐adherence will be essential for designing more effective behaviour‐centred strategies to improve the effectiveness of current regimens (Aunger & Curtis, 2016). RPs agreed with CPs regarding the need to assess current treatment guidelines but more from the perspective of proving that those treatments have low effectiveness (Abreha et al., 2017; Douglas et al., 2017; Maneeboonyang et al., 2011; Takeuchi et al., 2010) and thus strengthen the advocacy and business case for a policy change.

There are several limitations to this work. First, although APMEN serves as a unique forum for exchange between the research community and NMCPs, inevitable time restrictions prevent in‐depth discussions and more detailed analysis. CPs often have greater experience and capacity in policy implementation than in identifying and commissioning research to suit country needs.

Second, topics raised by RPs are shaped by their expertise and knowledge background, which is biased towards clinical and basic science with a lack of expertise in the fields of policy and health system research as well as limited participation by scientists with behavioural and social science backgrounds. This might also explain why participants did not raise topics around particular strategies to provide treatment to mobile populations and other hard‐to‐reach populations.

Third, the selection of research questions used for the ranking was done ad hoc at the end of the main meeting without considering the more detailed notes from each of the tables due to time limitations.

Fourth, there is a risk of potential power imbalance between RPs and CPs as RPs tended to be either native English speakers or have a very good working knowledge of English and were more confident in expressing their views. Whereas CPs may be less confident in expressing their views in English despite being highly knowledgeable in their country context. This results in CP voices often being heard less. To address this issue, roundtable discussions during the main meeting were separated for CPs and RPs and a follow‐up workshop was held exclusively for CPs.

Fifth, follow‐up interviews to verify some of the discussion outcomes were only held with three NMCPs. Interview participants were selected based on their availability within the timeframe of writing this article. Further in‐depth investigation is warranted to fully understand the evidence gaps.

Sixth, average priority scores across the region only have limited significance and given the heterogeneity between countries, localised research agendas need to be developed. In addition, individual CPs might not accurately represent country program priorities, but might be biased based on the specific area of work within the respective programs.

Seventh, the methods used to obtain frequency scores combined the average ranking of countries with roundtable scores to consider fewer CP than RP participants which could have skewed priorities towards those that were ranked.

Finally, we attempted to position research questions along a continuum of policy change while acknowledging that change is an iterative, non‐linear process that is influenced by many factors beyond evidence that require better understanding. As well as defining these evidence gaps, there is a need to understand the political economy of policy change processes. Providing data on ongoing research for CPs to draw from for timely decision‐making, packaging evidence in a manner that it translates clearly from RPs to CPs and continuing to strengthen the communication interface between RPs and CPs is also ideal. Work addressing these needs has already started and will inform policy change in the short and long term.

## 5 CONCLUSION

The safe and effective roll out of radical cure has repeatedly been identified by CPs as one of the major challenges of moving towards malaria elimination in vivax endemic countries (Thriemer et al., 2017). With the availability of novel tools and treatment strategies, the Asia‐Pacific target of elimination within the next 10 years appears more feasible. However, given the relatively short timeframe to achieve this 2030 target, the evidence gaps identified in our meetings need to be addressed urgently so that results become available at the same time as WHO provides a policy recommendation and revised global guidance on *P. vivax* treatment—anticipated in mid‐2021. This would enable NMCPs to examine global recommendations alongside nationally generated data relevant to their respective contexts.

## CONFLICT OF INTEREST

None declared.

## AUTHOR CONTRIBUTIONS

KT and RP conceived the overall meeting. KT, CAL, SN, EGG and VR planned and organised the workshop. VR collated data gathered by notetakers and facilitators at the meeting and the workshop. VR, MT, LD and MH contributed in‐depth data collection from Indonesia, Cambodia and Ethiopia. VR, KT, CAL and KP analysed the data. VR wrote the first draft of the article. KT, CAL, RP and KP have reviewed and edited the manuscript. All authors have read and approved the final manuscript.

## FUNDING INFORMATION

VR is funded by an Australian Government Research Training Program stipend from Charles Darwin University and an Australian Centre of Research Excellence in Malaria travel grant (APP 1134989). KT is funded by a CSL Centenary Fellowship, and a Bill and Melinda Gates Foundation grant (OPP1164105).

## Data Availability

The data that supports the findings of this study are available from the corresponding author upon reasonable request.

## APPENDIX A ROUNDTABLE ALLOCATION

**Table:**

Table	Facilitators	Note taker	NMCPs
CP table 1	Josselyn Neukom, APLMA	Rob Commons, Menzies School of Health Research	Afghanistan, Bhutan, Cambodia, India, Lao (PDR), Malaysia, Pakistan, PNG, Solomon Islands, Sri Lanka, Vanuatu, Ethiopia, China
CP table 2	Evelyn Wong, CHAI	Rittika Datta, APLMA	Afghanistan, Bhutan, China Indonesia, Lao (PDR), Nepal, Pakistan, Republic of Korea, Solomon Islands, Thailand, Vietnam, Ethiopia, PNG, Vanuatu
CP table 3	Spike Novak, PATH	Kemi Tesfezghi, PSI	Bangladesh, Cambodia, India, Indonesia, Malaysia, Nepal, Philippines, Republic of Korea, Sri Lanka, Thailand, Vietnam, Ethiopia
RP table 1	Nick Luter, PATH	Jutta Marfurt, Menzies School of Health Research	Researchers, endemic countries n = 6 Researchers, non‐endemic countries n = 3 PDP n = 1 WHO n = 1 Manufacturers n = 2
RP table 2	Angela Devine, Menzies School of Health Research	Sophie Weston, Menzies School of Health Research	Researchers, endemic countries n = 6 Researchers, non‐endemic countries n = 2 CSO n = 1 PDP n = 1 NMCP n = 1 Donor n = 1
RP table 3	Ros Howes, FIND	Varunika Ruwanpura, Menzies School of Health Research	Researchers, endemic countries n = 6 Researchers, non‐endemic countries n = 2 CSO n = 1 PDP n = 1 Donor n = 2
RP table 4	Sam McEvan, Burnet Institute	Chris Mercado, APMEN	Researchers, endemic countries n = 6 Researchers, non‐endemic countries n = 2 CSO n = 1 PDP n = 1 WHO n = 1 Manufacturers n = 1 NMCP n = 1
RP table 5	Melanie Larson, PATH	Kylie Mannion, APMEN	Researchers, endemic countries n = 4 Researchers, non‐endemic countries n = 4 CSO n = 1 Manufacturers n = 1 APMEN = 1
RP table 6	Vajra Allen, PATH	Yucheng Tsai, CHAI	Researchers, endemic countries n = 5 Researchers, non‐endemic countries n = 3 WHO n = 1 Manufacturers n = 1 APLMA n = 1

## APPENDIX B APMEN VxWG 2019 CP PARTICIPANTS

**Table:**

Country	Participant name
Afghanistan	Hamida Hamid
Afghanistan	Sami Nahzat
Bangladesh	MM Aktaruzzaman
Bhutan	Kinley Penjor
Bhutan	Rixin Jamtsho
Bhutan	Tobgyel Tobgyel
Cambodia	Chea Nguon
Cambodia	Lek Dysoley
China	Gao Qi
Ethiopia	Mebrahtom Haile Zeweli
India	Suman Lata Wattal
India	Sunil V Gittee
Indonesia	Minerva Theodora
Indonesia	Nurul Amin
Lao	Keobouphaphone Chindavongsa
Lao	Viengxay Vanisaveth
Lao	Vonethalom Thongpaseuth
Malaysia	Jenarun Jelip
Malaysia	Rose Nani Binti Mudin
Nepal	Anub Bastola
Nepal	Bibek Lal
Nepal	Ghanashyam Pokharel
Nepal	Kumar P Pokharel
Nepal	Leela Bikram Thapa
Nepal	Prakash P Sah
Nepal	Uttam Koirala
Nepal	Uttam Pyakurel
Nepal	Yuva Raj Pokharel
Pakistan	Abdul Majeed
Papua New Guinea	Leo Sora Makita
Philippines	Raffy Deray
Republic of Korea	Byoung Hak Jeon
Republic of Korea	Sang‐Eun Lee
Solomon Islands	Albino Bobogare
Solomon Islands	Leonard Boaz
Sri Lanka	K. D. N. P. Ranaweera
Sri Lanka	Megala Ravichandran
Thailand	Darin Areechokchai
Thailand	Prayuth Sudathip
Vanuatu	Esau Naket
Vietnam	Ngo Duc Thang
Vietnam	Nguyen Van Dung

Abbreviations: CPs, country partners; CSOs, Civil Society Organisations; PDPs, Product Development Partnerships; RPs, research partners.

*Note*: All CPs are leaders or members of NMCPs or the respective disease control divisions in their countries.

## APPENDIX C APMEN VxWG 2019 RP PARTICIPANTS

**Table:**

ACCESSBIO	Young S Hong
ACCESSBIO	BD Han
ACCESSBIO	Jacob Yang
APLMA, Singapore	Amita Chebbi
APLMA, Singapore	Josselyn Naukom
APLMA, Singapore	Rittika Datta
APLMA, Singapore	Vaibhav Gupta
APMEN, Singapore	Chris Mercado
APMEN, Singapore	Kylie Mannion
Australian National University, Australia	Kinley Wangdi
Burnet Institute, Australia	Jack Richards
Burnet Institute, Australia	Leanne Robinson
Burnet Institute, Australia	Rebecca Dabbs
Burnet Institute, Australia	Samuel McEwen
Clinton Health Access Initiative	Evelyn Wong
Clinton Health Access Initiative	Inessa Ba
Clinton Health Access Initiative	Yucheng Tsai
Clinton Health Access Initiative, Nepal	Kiran Awasti
Department of Foreign Affairs and Trade, Australia	Alyson Auliff
Eijkman Institute for Molecular Biology, Indonesia	Ari Satyagraha
Eijkman‐Oxford Clinical Research Unit, Indonesia	Kevin Baird
Ethiopian Public Health Institute, Ethiopia	Adugna Woyessa
Ethiopian Public Health Institute, Ethiopia	Ashenafi Assefa
FIND, Switzerland	Christian Ghergu
FIND, Switzerland	Rosalind Howes
GSK, UK	Andy Walker
icddr,b, Bangladesh	Wasif Khan
Institute of Cholera and Enteric Diseases, India	Arunansu Talukdar
Institute of Cholera and Enteric Diseases, India	Santasabuj Das
Institute of Tropical Medicine, Belgium	Charlotte Gryseels
Kasturba Medical College, India	Kavitha Saravu
London School of Hygiene & Tropical Medicine, UK	Henry Suhendra
Mahidol Vivax Research Center, Thailand	Wang Nguitragool
Mahidol Vivax Research Center, Thailand	Wanlapa Roobsoong
Malaria Atlas Project (MAP), UK	Katherine Battle
Malaria Medicines Venture (MMV), Switzerland	Melanie Larson
Malaria Medicines Venture (MMV), Switzerland	Caroline Lynch
Malaria Medicines Venture (MMV), Switzerland	Eric‐Marie Dupuy
Malaria Medicines Venture (MMV), Switzerland	George Jagoe
Menzies School of Health Research, Australia	Angela Devine
Menzies School of Health Research, Australia	Daniel Pfeffer
Menzies School of Health Research, Australia	Jutta Marfurt
Menzies School of Health Research, Australia	Kamala Thriemer
Menzies School of Health Research, Australia	Matthew Grigg
Menzies School of Health Research, Australia	Ric Price
Menzies School of Health Research, Australia	Robert Commons
Menzies School of Health Research, Australia	Sophie Weston
Menzies School of Health Research, Australia	Varunika Ruwanpura
Mahidol‐Oxford Research unit (MORU), Thailand	Cindy Chu
Nangarhar University, Afghanistan	Awab Ghulam Rahim
National Institute of Malariology, Parasitology and Entomology (NIMPE), Vietnam	Bui Quang Phuc
Oxford University Clinical Research Unit (OUCRU), Vietnam	Nguyen Chau
Oxford University, UK	Bipin Adhikari
Papua New Guinea Institute of Medical Research, PNG	Livingstone Tavul
Pasteur Institute, France	Narimane Nekkab
PATH, US	Nick Luter
PATH, US	Sampa Pal
PATH, US	Spike Nowak
PATH, US	Vajra Allan
Population Services International (PSI), Lao	Kemi Tesfazghi
Population Services International (PSI), Myanmar	Phone Si Hein
Research Institute for Tropical Medicine, Philippines	Effie Espino
Research Institute for Tropical Medicine, Philippines	Veronica L. Tallo
Retired Malaria Specialist, Nepal	Garib Das Thakur
Retired Malaria Specialist, Nepal	Padam B Chand
Save the Children, Nepal	Suman Thapa
SD Biosensor, Korea	Eun Kang Ye
Sukra Raj Tropical & Infectious Disease Hospital, Nepal	Basudev Pandey
Swedish Committee for Afghanistan, Afghanistan	Mohammad Anwar
Tribhuvan University, Nepal	Binad Dhungal
Tribhuvan University, Nepal	Jeevan B Sherchand
Tribhuvan University, Nepal	Keshab Parajuli
Tribhuvan University, Nepal	Komal Raj Rijal
Tribhuvan University, Nepal	Megha Raj Banjara
Tribhuvan University, Nepal	Naba Raj Adhikari
Tribhuvan University, Nepal	Prakash Ghimire
Tribhuvan University, Nepal	Upendra Thapa Shrestha
US President's Malaria Initiative/USAID, Myanmar	Nu Nu Khin
UCSF Global Health Group, US	Adam Bennett
Unitaid, Switzerland	Katerina Gallizzo
University of Gadjah Mada, Indonesia	Supargiyono Supargiyono
University of Indonesia, Indonesia	Erni Nelwan
University of Indonesia, Indonesia	Inge Sutanto
University of Melbourne, Australia	Julie Simpson
University of Sumatera Utara, Indonesia	Ayodhia Pitaloka
University of Sumatera Utara, Indonesia	Inke Nadia Diniyanti Lubis
University of Western Australia, Australia	Brioni Moore
University of Western Australia, Australia	Laurens Manning
Vector Borne Disease Research and Training Centre, Nepal	Yadu Chandra Ghimire
Walter and Eliza Hall Institute (WEHI), Australia	Robert James
Walter and Eliza Hall Institute (WEHI), Australia	Harin Karunajeewa
WHO, Nepal	Lungten Wangchuk
WHO, Nepal	Jos Vandealor
WHO, Switzerland	Jane Cunningham
WorldWide Antimalarial Resistance Network, UK	Philippe Guerin
YPKMP Timika, Indonesia	Annisa Rahmalia
YPKMP Timika, Indonesia	Rini Poespoprodjo

## APPENDIX D INITIAL EVIDENCE GAPS IDENTIFIED BY COUNTRY PARTNERS DURING THE MEETING'S ROUNDTABLE DISCUSSION

**Table:**

Discussion table number	Country partners	Identified research areas
1	Afghanistan, Bhutan, Cambodia, India, Lao (PDR), Malaysia, Pakistan, Papua New Guinea (PNG), Solomon Islands, Sri Lanka, Vanuatu, Ethiopia, Democratic People's Republic of Korea (DPRK), China	Operational research and policy implementation research
		Better knowledge translation and advocacy from research organisations for available evidence
		Better understanding of local data and context including G6PD prevalence in each country/Countries had different views regarding whether G6PD data should be population wide, on specific populations, or vivax specific.
		Further national studies relating to the global studies for radical cure options
		Comparison of efficacy, cost effectiveness, and safety of PQ7 versus PQ14 versus TQ
2	Afghanistan, Bhutan, China, Indonesia, Lao (PDR), Nepal, Pakistan, DPRK, Solomon Islands, Thailand, Vietnam, Republic of Korea (ROK), Ethiopia, PNG, Vanuatu	Better coordination between NMCPs and research institutes in countries
		Additional data and research on G6PD prevalence
		Efficacy and accuracy of qualitative and quantitative G6PD tests
		Research on addressing asymptomatic *P. vivax* malaria cases
		Research on the success of existing malaria control programmes
3	Bangladesh, Cambodia, India, Indonesia, Malaysia, Nepal, Philippines, Republic of Korea (ROK), Sri Lanka, Thailand, Vietnam, India	Technical evidence on efficacy of new diagnostic tests
		WHO recommendation on new drugs and the process for drug registration
		Operational feasibility based on level of health system, supply chain factors, human resource capacity and quality assurance procedures
		Cost benefit analysis for implementing radical cure and an assessment of the impact on national health budgets
		Drug acceptability: for patients and provider acceptability, this may require pilot studies.
		Development of a regional evidence base to influence national policy change

## APPENDIX F COUNTRY‐SPECIFIC RANKINGS OF RESEARCH PRIORITIES

### F.1 Afghanistan

**Table:**

Research question	Priority ranking
What is the prevalence of G6PD deficiency in my country?	1
What is the cost effectiveness of different regimen options?	2
What is the effectiveness of different options (PQ14, PQ7, TQ) and adherence between different regimens?	3
Feasibility of new interventions at different levels of health system (including supply chain capacity and quality assurance)?	4
What is the best way to improve patients' adherence?	5

### F.2 Bangladesh

**Table:**

Research question	Priority ranking
What is the prevalence of G6PD deficiency in my country?	1
How effective is the current practice?	2
What is the effectiveness of different options (PQ14, PQ7, TQ) and adherence between different regimens?	3
What is the best way to improve patients' adherence?	4
What is the cost effectiveness of different regimen options?	5

### F.3 Bhutan

**Table:**

Research question	Priority ranking
What is the prevalence of G6PD deficiency in my country?	1
What is the effectiveness of different options (PQ14, PQ7, TQ) and adherence between different regimens?	2
What is the cost effectiveness of different regimen options?	3
What are the overall vivax dynamics in my country?	4
How can we ensure safe delivery of the different options?	5

### F.4 Cambodia

**Table:**

Research question	Priority ranking
What is the effectiveness of different options (PQ14, PQ7, TQ) and adherence between different regimens?	1
What is the cost effectiveness of different regimen options?	2
How can we ensure safe delivery of the different options?	3
How well do new diagnostics work in the field (usability, robustness, etc.)?	4
Feasibility of new interventions at different levels of health system (including supply chain capacity and quality assurance)?	5

### F.5 Ethiopia

**Table:**

Research question	Priority ranking
What is the best way to improve patients' adherence?	1
How can we ensure safe delivery of the different options?	2
What is the prevalence of G6PD deficiency in my country?	3
What is the cost effectiveness of different regimen options?	4
How well do new diagnostics work in the field (usability, robustness, etc.)?	5

### F.6 India

**Table:**

Research question	Priority ranking
What is the effectiveness of different options (PQ14, PQ7, TQ) and adherence between different regimens?	1
How well do new diagnostics work in the field (usability, robustness, etc.)?	2
How can we ensure safe delivery of the different options?	3
What is the best way to improve patients' adherence?	4
What is the cost effectiveness of different regimen options?	5

### F.7 Indonesia

**Table:**

Research question	Priority ranking
Feasibility of new interventions at different levels of health system (including supply chain capacity and quality assurance)?	1
How well do new diagnostics work in the field (usability, robustness, etc.)?	2
How can we ensure safe delivery of the different options?	3
What is the cost effectiveness of different regimen options?	4
What is the effectiveness of different options (PQ14, PQ7, TQ) and adherence between different regimens?	5

### F.8 Lao (PDR)

**Table:**

Research question	Priority ranking
What is the effectiveness of different options (PQ14, PQ7, TQ) and adherence between different regimens?	1
How well do new diagnostics work in the field (usability, robustness, etc.)?	2
How can we ensure safe delivery of the different options?	3
What is the best way to improve patients' adherence?	4
Feasibility of new interventions at different levels of health system (including supply chain capacity and quality assurance)?	5

### F.9 Malaysia

**Table:**

Research question	Priority ranking
How effective is the current practice?	1
What is the cost effectiveness of different regimen options?	2
How well do new diagnostics work in the field (usability, robustness, etc.)?	3
Feasibility of new interventions at different levels of health system (including supply chain capacity and quality assurance)?	4
Not answered	5

### F.10 Nepal

**Table:**

Research question	Priority ranking
What is the best way to improve patients' adherence?	1
How well do new diagnostics work in the field (usability, robustness, etc.)?	2
How effective is the current practice?	3
What is the effectiveness of different options (PQ14, PQ7, TQ) and adherence between different regimens?	4
Feasibility of new interventions at different levels of health system (including supply chain capacity and quality assurance)?	5

### F.11 Pakistan

**Table:**

Research question	Priority ranking
What are the overall vivax dynamics in my country?	1
What is the prevalence of G6PD deficiency in my country?	2
What is the effectiveness of different options (PQ14, PQ7, TQ) and adherence between different regimens?	3
How well do new diagnostics work in the field (usability, robustness, etc.)?	4
What is the best way to improve patients' adherence?	5

### F.12 Papua New Guinea (PNG)

**Table:**

Research question	Priority ranking
What is the prevalence of G6PD deficiency in my country?	1
What is the best way to improve patients' adherence?	2
Feasibility of new interventions at different levels of health system (including supply chain capacity and quality assurance)?	3
What is the cost effectiveness of different regimen options?	4
What are the overall vivax dynamics in my country?	5

### F.13 Solomon Islands

**Table:**

Research question	Priority ranking
What is the effectiveness of different options (PQ14, PQ7, TQ) and adherence between different regimens?	1
Feasibility of new interventions at different levels of health system (including supply chain capacity and quality assurance)?	2
How well do new diagnostics work in the field (usability, robustness, etc.)?	3
What is the best way to improve patients' adherence?	4
How effective is the current practice?	5

### F.14 Republic of Korea

**Table:**

Research question	Priority ranking
What is the effectiveness of different options (PQ14, PQ7, TQ) and adherence between different regimens?	1
Not answered	2
Not answered	3
Not answered	4
Not answered	5

### F.15 Sri Lanka

**Table:**

Research question	Priority ranking
What is the effectiveness of different options (PQ14, PQ7, TQ) and adherence between different regimens?	1
Feasibility of new interventions at different levels of health system (including supply chain capacity and quality assurance)?	2
How well do new diagnostics work in the field (usability, robustness, etc.)?	3
How can we ensure safe delivery of the different options?	4
What is the cost effectiveness of different regimen options?	5

### F.16 Thailand

**Table:**

Research question	Priority ranking
What is the effectiveness of different options (PQ14, PQ7, TQ) and adherence between different regimens?	1
What is the best way to improve patients' adherence?	2
Feasibility of new interventions at different levels of health system (including supply chain capacity and quality assurance)?	3
How can we ensure safe delivery of the different options?	4
How well do new diagnostics work in the field (usability, robustness, etc.)?	5

### F.17 Vanuatu

**Table:**

Research question	Priority ranking
What is the effectiveness of different options (PQ14, PQ7, TQ) and adherence between different regimens?	1
How well do new diagnostics work in the field (usability, robustness, etc.)?	2
What is the best way to improve patients' adherence?	3
How can we ensure safe delivery of the different options?	4
What is the cost effectiveness of different regimen options?	5

### F.18 Vietnam

**Table:**

Research question	Priority ranking
What is the effectiveness of different options (PQ14, PQ7, TQ) and adherence between different regimens?	1
What is the cost effectiveness of different regimen options?	2
Feasibility of new interventions at different levels of health system (including supply chain capacity and quality assurance)?	3
How well do new diagnostics work in the field (usability, robustness, etc.)?	4
What is the prevalence of G6PD deficiency in my country?	5
